# Human Activity Recognition Using Gaussian Mixture Hidden Conditional Random Fields

**DOI:** 10.1155/2019/8590560

**Published:** 2019-08-18

**Authors:** Muhammad Hameed Siddiqi, Madallah Alruwaili, Amjad Ali, Saad Alanazi, Furkh Zeshan

**Affiliations:** ^1^College of Computer and Information Sciences, Jouf University, Sakaka, Saudi Arabia; ^2^Department of Computer Science, COMSATS University Islamabad, Lahore Campus, Lahore, Pakistan

## Abstract

In healthcare, the analysis of patients' activities is one of the important factors that offer adequate information to provide better services for managing their illnesses well. Most of the human activity recognition (HAR) systems are completely reliant on recognition module/stage. The inspiration behind the recognition stage is the lack of enhancement in the learning method. In this study, we have proposed the usage of the hidden conditional random fields (HCRFs) for the human activity recognition problem. Moreover, we contend that the existing HCRF model is inadequate by independence assumptions, which may reduce classification accuracy. Therefore, we utilized a new algorithm to relax the assumption, allowing our model to use full-covariance distribution. Also, in this work, we proved that computation wise our method has very much lower complexity against the existing methods. For the experiments, we used four publicly available standard datasets to show the performance. We utilized a 10-fold cross-validation scheme to train, assess, and compare the proposed model with the conditional learning method, hidden Markov model (HMM), and existing HCRF model which can only use diagonal-covariance Gaussian distributions. From the experiments, it is obvious that the proposed model showed a substantial improvement with *p* value ≤0.2 regarding the classification accuracy.

## 1. Introduction

In real-life environments, there are some fascinating applications in which the analysis of human activities plays a significant role. Some applications include human/object detection and recognition based on vision object analysis and processing areas such as tracking and detection [1, 2], computer engineering [3], physical sciences [4], health-related issues, natural sciences, and industrial academic areas [5]. Most of the authors [6–11] recognized the human activities in indoor environments based on different methodologies. However, in their respective systems, they used stable environment like fixed camera setting and prelighting setting, and most of the activities were performed by the instructions provided by the instructor. Similarly, the authors of [10, 12–14] proposed different methods to recognize the human daily activities in outdoor environments. However, in most of the used datasets, they used static background and this is one of the common drawbacks in their systems. Similarly, different sensors were utilized by the authors of [15–17] in order to classify indoor and outdoor human activities.

Moreover, in telemedicine and healthcare, human activity recognition (HAR) can be explained by helping physically disabled persons' scenario. A paralyzed patient with half of the body critically disturbed by stroke is completely unable to walk and the one way to recover him is through daily exercises. Normally, the daily exercises (activities) are recommended by the doctors to the stroke patients for getting better improvements in their health. A human activity recognition (HAR) system can correctly train and identify the activities performed by the stroke patients, through which the doctors easily can monitor the improvement scale in the patients' health.

**Figure 1:**
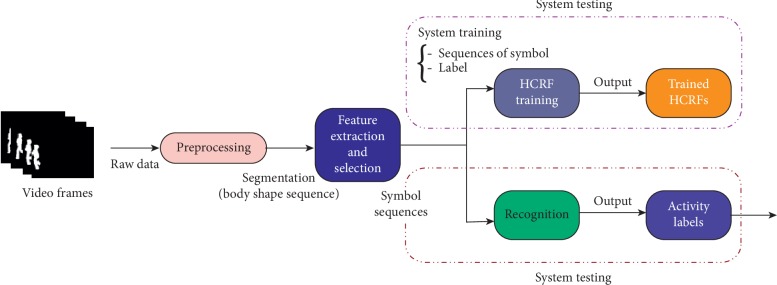
A typical human activity recognition (HAR) system.

There are four modules in a typical HAR system: preprocessing (segmentation), feature extraction, feature selection, and recognition as shown in Figure 1. Most of the existing works [18–23] focused on feature extraction and selection; however, very limited works have been done for the recognition module. Some studies exploited conventional techniques [24–28]. Among them, HMM is one of the best candidates for the activity recognition; however, HMM is generative in nature and less precise than its matching part like HCRF model [29].

The inspiration behind the recognition stage is the lack of enhancement in the learning method. Therefore, we have made the following contribution:

- The existing HCRF model is inadequate by independence assumptions, which may reduce classification accuracy. Therefore, the first objective of this study is to propose a recognition model that presents a new algorithm to relax the assumption, allowing our model in order to use full -covariance distribution.
- Another objective of the work is to prove that computation wise our method has very much lower complexity against the existing methods. In this method, our goal was to find some parameters to maximize the conditional probability of the training data at the training phase. Therefore, in our work, we utilize limited-memory Broyden–Fletcher–Goldfarb–Shanno (L-BFGS) method to search for the optimal point. However, instead of repeating the forward and backward algorithms to compute the gradients as others did [30], we run the forward and backward algorithms only when calculating the conditional probability, and then we reuse the result to compute the gradients. As a result, the computation time is significantly reduced.
- A comprehensive set of experiments which yielded a weighted average classification rate 97% that is better improvement in the performance against the state-of-the-art methods.

The rest of the paper is organized as follows: Section 2 presents related works with their limitations.  Section 3 provides the proposed recognition model with its advantages.  Section 4 describes the experimental setup for the proposed model against four datasets. Based on the setup, a series of experiments are presented in Section 5. Finally, Section 6 describes conclusion with some future directions.

## 2. Related Works

In a typical HAR system, different types of latest segmentation methods were used in preprocessing module in order to extract the human body from the activity frame. This process helps to improve the performance of the activity recognition system. Therefore, in the literature, the authors of [31–36] utilized the latest methods to segment the human body from the video frames. Similarly, for the feature extraction, different latest methodologies have been employed which help the classifiers to accurately classify the human activities (as the workflow shown in Figure 1) [37–42]. They showed better performance on different datasets, and most of them achieved average accuracy between 70 and 90%.

Regarding recognition, the researchers have proposed diverse systems which exploit various classifiers such as Gaussian mixture model (GMM) [43, 44], artificial neural network (ANN) [45, 46], and support vector machine (SVMs) [47–50]. These classifiers were principally employed for frame-based classification. Contrarily, in many HAR systems [37, 51, 52], the eminent hidden Markov model (HMM) has extensively been utilized for sequence-based classification. In the case of frame-level features, HMMs are benefited over vector-based classifiers like SVM, GMM, and ANN in terms of effectively handling the sequential data. However, the Markovian property implied in the traditional HMM assumes that the current state is a function of the past state only. This causes the labels of two adjacent states in the observation sequence to hypothetically appear in succession. But in practical implementation, this assumption often does not meet satisfaction. Besides, the generative characteristic of HMM and independence presumptions between observations and states also limit its performance [29]. To get rid of these limitations, the maximum entropy Markov model (MEMM) had been proposed which comparatively performs better than HMM [53]. However, MEMM is associated with the well-known disadvantage termed as “*label bias problem*”.

Two generalized models of MEMM known as conditional random fields (CRFs) [29] and HCRF [54] were developed to fix the shortcoming of “*label bias problem*” [29]. For learning the hidden structure of the sequential data, HCRF facilitates the effectiveness of CRF with hidden states. However, in both models, the per-state normalization is replaced with global normalization, permitting the weighted scores which in turn result in larger parameter spaces as compared to HMM and MEMM.

For example, the CRFs achieved in the HAR system having the observed frames from a video are represented by feature vector *U*, resultant label *V*, and unknown state label *K*.

Suppose, the problem image labeling is assumed by original labels *K* with image features *U* and parameter of the model is Λ, then the later probability (post(*K* | *U*; Λ)) maximized by CRF is given as

(1) $$\begin{array}{c} \text{post}\left(K\;|\;U;\Lambda \right)=\frac{e_{x}\left(f\left(K,U,\Lambda \right)\right)}{z\left(U,\Lambda \right)}, \end{array}$$

where the normalization factor is

(2) $$\begin{array}{c} z\left(U,\Lambda \right)=\underset{K^{\prime }}{\sum }e_{x}\left(f\left(K^{\prime },U,\Lambda \right)\right). \end{array}$$

Some issues in HCRF implementation are reviewed and analyzed in the following description. The later probability of CRF in (1) has been updated by the post(*K* | *U*; Λ) in a HCRF model that is the addition of exponentials of latent functions with all expected labels *L* as given below

(3) $$\begin{array}{c} \text{post}\left(V\;|\;U;\Lambda \right)=\frac{\sum_{\bar{L}}e_{x}\left\{\Lambda \cdot f\left(V,\bar{L},U\right)\right\}}{z\left(U,\Lambda \right)},\\ z\left(U,\Lambda \right)=\underset{V^{\prime }\bar{L}}{\sum }e_{x}\left\{\Lambda \cdot f\left(V^{\prime },\bar{L},U\right)\right\}. \end{array}$$

The above equations are used to warranty the sum to one rule of the conditional probability. *V*′ is the possible tag for the series of frames, and $\bar{L}=\left\{l_{1},l_{2},\ldots ,l_{T}\right\}$ is a series of hidden states *l*_*i*_,  *i* = 1,2,…, *T*, and equations (1) and (2) have constant values from 1 to Q (the number of states), *Λ* is the vector factor, and $f\left(V,\bar{L},U\right)$ is a feature vector that will yield a decision which parameter will be educated by the model. Then, the feature vector concludes the addition of the existing HCRF model. For example, the underneath selections will create a Markov restraint HCRF with a Gaussian distribution at every state:

(4) $$\begin{array}{c} f_{l}^{\text{Prior}}\left(V,\bar{L},U\right)=\delta \left(l_{1}=l\right)\cdot v,\;u\forall l,\\ f_{ll^{\prime }}^{\text{Transition}}\left(V,\bar{L},U\right)=\overset{T}{\underset{t=1}{\sum }}\delta \left(l_{t-1}=l\right)\delta \left(l_{t}=l^{\prime }\right)\cdot v,\;u\forall l,l^{\prime },\\ f_{l}^{\text{Occurence}}\left(V,\bar{L},U\right)=\overset{T}{\underset{t=1}{\sum }}\delta \left(l_{t}=l\right)\cdot v,\;u\forall l, \end{array}$$

where each *v* ∈ *V* is the expected tag and every *u* ∈ *U* is a predicted vector. The per-component square of the observation vector *v* at state *t* (i.e., *v*_*t*_) is given as

(5) $$\begin{array}{c} f_{l}^{M1}=\overset{T}{\underset{t=1}{\sum }}\delta \left(l_{t}=l\right)\cdot v,\;u_{t}\forall l,\\ f_{l}^{M2}\left(V,\bar{L},U\right)=\overset{T}{\underset{t=1}{\sum }}\delta \left(l_{t}=l\right)\cdot v,\;u_{t}^{2}\forall l. \end{array}$$

It can be seen that along with certain set of parameters (*Λ*), the HCRF addition is similar to the hidden Markov model, for instance along with the abovementioned feature vector, if we choose

(6) $$\begin{array}{c} \overset{\text{Prior}}{\underset{l}{\Lambda }}=\text{log}\left(b\left(l\right)\right) \end{array}$$

(7) $$\begin{array}{c} \overset{\text{Transition}}{\underset{l}{\Lambda }}=\log\left(C\left(l,l^{\prime }\right)\right), \end{array}$$

(8) $$\begin{array}{c} \overset{\text{Occurence}}{\underset{l}{\Lambda }}=-\frac{1}{2}\left(\text{log}\left(2\pi \sigma_{l}^{2}\right)+\frac{\mu_{l}^{2}}{\sigma_{l}^{2}}\right), \end{array}$$

(9) $$\begin{array}{c} \Lambda_{l}^{M1}=\frac{\mu_{l}}{\sigma_{l}^{2}}, \end{array}$$

(10) $$\begin{array}{c} \Lambda_{l}^{M2}=-\frac{1}{2\sigma_{l}^{2}}, \end{array}$$

where *b* in (6) is an earlier dissemination of Gaussian HMM and *C* in (7) is an evolution matrix; then conditional possibility numerator might be explained as

(11) $$\begin{array}{c} \underset{\bar{L}}{\sum }e_{x}\left\{\Lambda \cdot f\left(V,\bar{L},U\right)\right\}=\underset{\bar{L}}{\sum }b\left(l_{1}\right)\overset{T}{\underset{t=1}{\prod }}C\left(l_{t-1},l_{t}\right)N\left(u_{t}^{2},\mu_{L_{t}},\sigma_{L_{t}}\right). \end{array}$$

In the above equation, *N* represents Gaussian distribution. Equation (11) is the conditional probability of *U*, given *V* is calculated along with a Gaussian HMM through equation (11) which has an earlier distribution *b* with a conversion matrix *C*.

Moreover, the authors of [30] proposed a comprehensive form of the HCRF model to tackle composite scatterings utilizing a linear combination of Gaussian distribution functions, which is explained as

(12) $$\begin{array}{c} \text{post}\left(V\;|\;U;\Lambda \right)=\frac{\sum_{\bar{L}}\sum_{m=1}^{M}e_{x}\left\{\Lambda f\left(V,\bar{L},m,U\right)\right\}}{z\left(U,\Lambda \right)}. \end{array}$$

In equation (12), *M* indicates the number of components in Gaussian mixture.

Lots of works have been developed which showed better performance based on the usage of the abovementioned HCRF [55, 56]; however, most of them did not consider the limitations of the model. It is obvious from the aforementioned equations that the existing model employed diagonal (sloping)-covariance Gaussian distribution, which means that the variables (columns of *u*_*i*_,  *i* = 1,2,…, *N*) were presumed to be couples independent. On the other hand, equations (8)–(10) suggest that with a specific set of value, each state observation density will congregate to Gaussian procedure. Unluckily, there is no training method designed yet to guarantee this convergence, and those suppositions might decrease the accuracy results.

Therefore, we proposed the improved version of the HCRF technique that has the ability to openly employ full-covariance Gaussian mixture in the feature function. The proposed model will get the benefits of hidden conditional random field model that completely considered the drawbacks of the previous method.

## 3. Proposed Methodology

### 3.1. Feature Extraction

In our previous work, we utilized symlet wavelet [37] for extracting various features from the activity frames. There are number of reasons for using the symlet wavelet which produces relatively better classification results. These include its capability to extract the conspicuous information from the activity frames in terms of frequency and its support to the characteristics of the grayscale images like orthogonality, biorthogonality, and reverse biorthogonality. For a certain provision size, the symlet is characterized with the highest number of vanishing moments and has the least asymmetry.

### 3.2. Proposed Hidden Conditional Random Fields (HCRFs) Model

As described earlier, the current Gaussian mixture HCRF model does not have the capability of utilizing full-covariance distributions and also does not guarantee the conjunction of its factors to certain values upon which the conditional probability is demonstrated as a combination of the normal density functions.

To address these limitations, we explicitly involve a mixture of Gaussian distributions in the feature functions as illustrated in the following forms:

(13) $$\begin{array}{c} \overset{\text{Prior}}{\underset{l}{f}}\left(V,\bar{L},U\right)=\delta \left(l_{1}=l\right)\cdot v,\;u\forall l, \end{array}$$

(14) $$\begin{array}{c} \overset{\text{Transition}}{\underset{ll^{\prime }}{f}}\left(V,\bar{L},U\right)=\overset{T}{\underset{t=1}{\sum }}\delta \left(l_{t-1}=l\right)\delta \left(l_{t}=l^{\prime }\right)\cdot v,\;u\forall l,l^{\prime }, \end{array}$$

(15) $$\begin{array}{c} \overset{\text{Observation}}{\underset{l}{f}}\left(V,\bar{L},U\right)=\overset{T}{\underset{t=1}{\sum }}\log\left(\overset{M}{\underset{m=1}{\sum }}\Gamma_{l,m}^{\text{Obs}}N\left(u_{t}^{2},\mu_{l,m},\Sigma_{l,m}\right)\right)\delta \left(l_{t}=l\right)\cdot v, \end{array}$$

then,

(16) $$\begin{array}{c} N\left(u_{t}^{2},\mu_{l,m},\Sigma_{l,m}\right)=\frac{1}{{\left(2\pi \right)}^{D/2}{\left|\Sigma_{l,m}\right|}^{1/2}}e_{x}\left(-\frac{1}{2}{\left(u_{t}^{2}-\mu_{l,m}\right)}^{\prime }\overset{-1}{\underset{l,m}{\sum }}\left(x_{t}^{2}-\mu_{l,m}\right)\right), \end{array}$$

where *N* represents the number of density functions, Gamma “Γ” considers the appropriate information of the entire observations, *D* indicates the dimension of the observation, and Γ_*l*,*m*_^Obs^ presents the partying weightiness for the *m* ^th^ constituent along with mean *μ*_*l*,*m*_ and covariance matrix *Σ*_*l*,*m*_.

As indicated in equation (14), when we change some of the parameters such as Γ, *μ*, and Σ, then we may build a combination of the standard densities. The resultant conditional probability might be written as

(17) $$\begin{array}{c} \text{post}\left(V\;|\;U;\Lambda ,\Gamma ,\mu ,\Sigma \right)=\frac{\sum_{\bar{L}}e_{x}\left(P\left(\bar{L}\right)+T\left(\bar{L}\right)+O\left(\bar{L}\right)\right)}{z\left(U,\Lambda ,\Gamma ,\mu ,\Sigma \right)},\\ P\left(\bar{L}\right)=\underset{l}{\sum }\Lambda_{l}^{\text{Prior}}f_{l}^{\text{Prior}}\left(V,\bar{L},U\right),\\ T\left(\bar{L}\right)=\underset{ll^{\prime }}{\sum }\Lambda_{ll^{\prime }}^{\text{Transition}}f_{ll^{\prime }}^{\text{Transition}}\left(V,\bar{L},U\right),\\ O\left(\bar{L}\right)=\underset{l}{\sum }f_{l}^{\text{Observation}}\left(V,\bar{L},U\right), \end{array}$$

therefore,

(18) $$\begin{array}{c} \text{post}\left(V\;|\;U;\Lambda ,\Gamma ,\mu ,\Sigma \right)=\frac{\sum_{\bar{L}}\text{exp}\left(\sum_{l}\Lambda_{l}^{Prior}f_{l}^{Prior}\left(V,\bar{L},U\right)+\sum_{ll^{\prime }}\Lambda_{ll^{\prime }}^{\text{Transition}}f_{ll^{\prime }}^{\text{Transition}}\left(V,\bar{L},U\right)+\sum_{l}f_{l}^{\text{Observation}}\left(V,\bar{L},U\right)\right)}{z\left(U,\Lambda ,\Gamma ,\mu ,\Sigma \right)}, \end{array}$$

(19) $$\begin{array}{c} \text{post}\left(V\;|\;U;\Lambda ,\Gamma ,\mu ,\Sigma \right)=\frac{\sum_{\bar{L}=l_{1},l_{2},..,l_{T}}e_{x}\left(\Lambda_{l_{1}}^{\text{Prior}}+\sum_{t=1}^{T}\left(\Lambda_{l_{t-1},l_{t}}^{\text{Transition}}\right)+\text{log}\left(\sum_{m=1}^{M}\Gamma_{l_{t},m}^{\text{Obs}}N\left(u_{t}^{2},\mu_{l_{t},m},\Sigma_{l_{t},m}\right)\right)\right)}{z\left(U,\Lambda ,\Gamma ,\mu ,\Sigma \right)}, \end{array}$$

(20) $$\begin{array}{c} \text{post}\left(V\;|\;U;\Lambda ,\Gamma ,\mu ,\Sigma \right)=\frac{\text{Score}\left(\left.U\right|V;\Lambda ,\Gamma ,\mu ,\Sigma \right)}{z\left(U;\Lambda ,\Gamma ,\mu ,\Sigma \right)}. \end{array}$$

The forward and backward algorithms are used to calculate the conditional probability based on equations (19) and (20) that can be written as

(21) $$\begin{array}{c} \alpha_{\tau }=\underset{\bar{L}=l_{1},l_{2},..,\left\{l_{\tau }=l\right\}}{\sum }\text{exp}\left(\Lambda_{l_{1}}^{\text{Prior}}+\overset{T}{\underset{t=1}{\sum }}\left(\Lambda_{l_{t-1},l_{t}}^{\text{Transition}}\right)+\text{log}\left(\overset{M}{\underset{m=1}{\sum }}\Gamma_{l_{t},m}^{\text{Obs}}N\cdot \left(u_{t}^{2},\mu_{l_{t},m},\Sigma_{l_{t},m}\right)\right)\right)\\ =\underset{l\prime }{\sum }\alpha_{\tau -1}\left(l^{\prime }\right)\text{exp}\left(\Lambda_{ll^{\prime }}^{\text{Transition}}+\text{log}\overset{M}{\underset{m=1}{\sum }}\Gamma_{l_{t},m}^{\text{Obs}}N\cdot \left(u_{\tau },\mu_{l,m},\Sigma_{l,m}\right)\right), \end{array}$$

(22) $$\begin{array}{c} \beta_{\tau }\left(l\right)=\underset{\bar{L}=\left\{l_{\tau }=l\right\},l_{\tau +1},\ldots ,l_{T}}{\sum }e_{x}\left(\Lambda_{l_{1}}^{\text{Prior}}+\overset{T}{\underset{t=1}{\sum }}\left(\Lambda_{l_{t-1},l_{t}}^{\text{Transition}}\right)+\text{log}\overset{M}{\underset{m=1}{\sum }}\Gamma_{l_{t},m}^{\text{Obs}}N\cdot \left(u_{t}^{2},\mu_{l_{t},m},\Sigma_{l_{t},m}\right)\right)\\ =\underset{l\prime }{\sum }\beta_{\tau +1}\left(l^{\prime }\right)\text{exp}\left(\Lambda_{ll^{\prime }}^{\text{Transition}}+\text{log}\left(\overset{M}{\underset{m=1}{\sum }}\Gamma_{l,m}^{\text{Obs}}N\left(u_{\tau },\mu_{l,m},\Sigma_{l,m}\right)\right)\right), \end{array}$$

(23) $$\begin{array}{c} \text{Score}\left(V\;|\;V;\Lambda ,\Gamma ,\mu ,\Sigma \right)=\underset{l}{\sum }\alpha_{T}\left(l\right)=\underset{l}{\sum }\beta_{1}\left(l\right). \end{array}$$

In the training data, to maximize the conditional probability, we initially focused on calculating the parameters (Λ, Γ, *μ*, and Σ). In the proposed approach, limited-memory Broyden–Fletcher–Goldfarb–Shanno (L-BGFS) method has been implemented in order to search the optimum point. Unlikely the other models [30], both the forward and backward algorithms are used to compute the conditional probability and the results were reused for finding the gradients. This makes the algorithm more significant in reducing the computation time.

At the observation level, we particularly incorporated the full-covariance matrix in the feature function as shown in (16). Equation (17) may be used for getting the normal distribution which is further elaborated in the following equations:

(24) $$\begin{array}{c} \frac{d\text{ Score}\left(V\;|\;U;\Lambda ,\Gamma ,\mu ,\Sigma \right)}{d\Lambda_{l}^{\text{Prior}}}=\underset{\bar{L}}{\sum }\frac{dg\left(V,\bar{L},U\right)}{d\Lambda_{l}^{\text{Prior}}}e_{x}\left(g\left(V,\bar{L},U\right)\right)\\ =\underset{\bar{L}}{\sum }f_{l}^{\text{Prior}}\left(V,\bar{L},U\right)e_{x}\left(g\left(V,\bar{L},U\right)\right)=\beta_{1}\left(l\right). \end{array}$$

The *d* Score function is a gradient function for a variable of the prior probability vector:

(25) $$\begin{array}{c} \frac{d\text{ Score}\left(V\;|\;U;\Lambda ,\Gamma ,\mu ,\Sigma \right)}{d\Lambda_{l}^{\text{Transition}}}=\underset{\bar{L}}{\sum }\frac{dg\left(V,\bar{L},U\right)}{d\Lambda_{ll^{\prime }}^{\text{Transition}}}e_{x}\left(g\left(V,\bar{L},U\right)\right)\\ =\underset{\bar{L}}{\sum }f_{ll^{\prime }}^{\text{Transition}}\left(V,\bar{L},U\right)\text{exp}\left(g\left(V,\bar{L},U\right)\right)=\overset{T}{\underset{t=1}{\sum }}\alpha \left(t,l\right)\beta \left(t+1,l\prime \right). \end{array}$$

The *d* Score function is a gradient function for a variable of the transition probability vector:

(26) $$\begin{array}{c} \frac{d\text{ Score}\left(V\;|\;U;\Lambda ,\Gamma ,\mu ,\Sigma \right)}{d\Gamma_{l,m}^{\text{Obs}}}=\underset{\bar{L}}{\sum }\frac{dg\left(V,\bar{L},U\right)}{d\Gamma_{l,m}^{\text{Obs}}}e_{x}\left(g\left(V,\bar{L},U\right)\right)\\ =\frac{\sum_{\bar{L}}f_{l}^{\text{Observation}}\left(V,\bar{L},U\right)}{d\Gamma_{l,m}^{\text{Obs}}}e_{x}\left(g\left(V,\bar{L},U\right)\right)\\ =\underset{\bar{L}}{\sum }\overset{T}{\underset{t=1}{\sum }}\frac{N\left(u_{t},\mu_{l,m},\Sigma_{l,m}\right)}{\sum_{m=1}^{M}\Gamma_{l,m}^{\text{Obs}}N\left(u_{t},\mu_{l,m},\Sigma_{l,m}\right)}\delta \left(l_{t}=l\right)e_{x}\left(g\left(V,\bar{L},U\right)\right)\\ =\overset{T}{\underset{t=1}{\sum }}\frac{N\left(u_{t},\mu_{l,m},\Sigma_{l,m}\right)}{\sum_{m=1}^{M}\Gamma_{l,m}^{\text{Obs}}N\left(u_{t},\mu_{l,m},\Sigma_{l,m}\right)}\alpha \left(t,l\right)\gamma \left(t+1\right). \end{array}$$

The *d* Score function is a gradient function for a Gaussian mixture weight variable. Here, a function *V*(*t*) can be determined as

(27) $$\begin{array}{c} \gamma \left(t\right)=\underset{l}{\sum }\beta \left(t,l\right), \end{array}$$

(28) $$\begin{array}{c} \frac{d\text{ Score}\left(V\;|\;U;\Lambda ,\Gamma ,\mu ,\Sigma \right)}{d\mu_{l,m}}=\overset{T}{\underset{t=1}{\sum }}\frac{\Gamma_{l,m}^{\text{Obs}}dN\left(u_{t},\mu_{l,m},\Sigma_{l,m}\right)/d\mu_{l,m}}{\sum_{m=1}^{M}\Gamma_{l,m}^{\text{Obs}}N\left(u_{t},\mu_{l,m},\Sigma_{l,m}\right)}\alpha \left(t,l\right)\gamma \left(t+1\right). \end{array}$$

The *d* Score function is a gradient function for the Gaussian distribution mean:

(29) $$\begin{array}{c} \frac{d\text{ Score}\left(V\;|\;U;\Lambda ,\Gamma ,\mu ,\Sigma \right)}{d\Sigma_{l,m}}=\overset{T}{\underset{t=1}{\sum }}\frac{\Gamma_{l,m}^{\text{Obs}}dN\left(u_{t},\mu_{l,m},\Sigma_{l,m}\right)/d\Sigma_{l,m}}{\sum_{m=1}^{M}\Gamma_{l,m}^{\text{Obs}}N\left(u_{t},\mu_{l,m},\Sigma_{l,m}\right)}\alpha \left(t,l\right)\gamma \left(t+1\right). \end{array}$$

The *d* Score function is a gradient function for the covariance of the Gaussian distributions.

Equations (24)–(27) presented above describe an analysis method algorithm for calculating values of gradients for a feature function, the mean of Gaussian distributions, and the covariance of the prior probability vector, the transition probability vector, and the observation probability vector obtained from the existing HCRF.

In our model, the recognition of a variety of real-time activities can be divided into two steps: a training step and an inference step. In the first step, data with known labels are inputted for recognizing the target as well as training the hidden conditional field model. In the inference step, the inputs to be actually estimated are ordered dependent on parameters determined in the training phase.

If the activity frame is acting as an input in the training step, then, in the preprocessing step, the applied distinctive lighting effects are decreased for detecting and extracting faces from the activity frames. At that point, the movable features are extricated from the various facial parts for creating the feature vector. After that, the feature vector obtained serves as an input to a full-covariance Gaussian-mixed hidden conditional random field model of the suggested recognition model.

**Figure 2:**
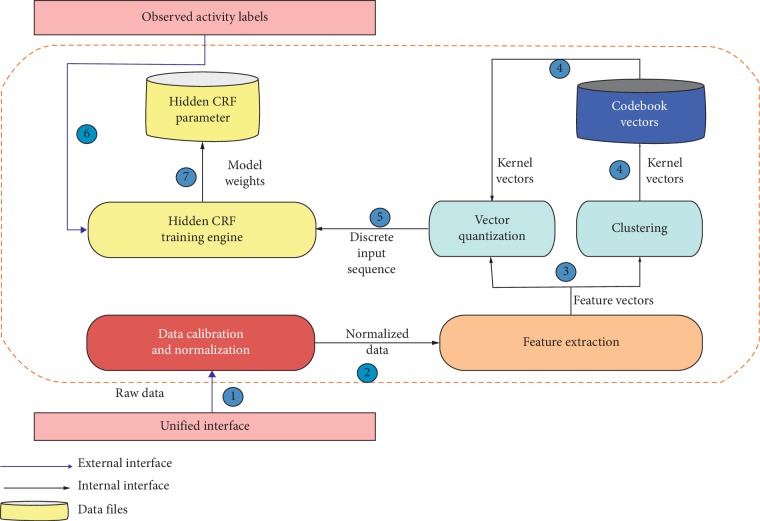
Workflow diagram of the proposed recognition model.

As mentioned in the earlier discussion, a feature gradient is generally determined by LBFG approach in the training phase of the HCRF model. Nonetheless, in the current gradient calculation technique, a forward and backward iterative execution algorithm is iteratively called upon, which needs an exceptionally high computational time and thus leads to reduction in the computational speed. Another analysis approach has been formulated that reduces the invoking of the forward and backward iterative execution algorithm using five gradient functions determined by equations (24)–(28). Using this analysis, the real-time computation can be carried out at a higher speed resulting in an enormous decrease in the computational time compared to a known analysis approach. The overall workflow of the proposed model is shown in Figure 2.

## 4. Model Validation

### 4.1. Datasets Used

In this work, we employed four open-source standard action datasets like Weizmann action datasets [57], KTH action dataset [58], UCF sports dataset [59], and IXMAS action dataset [60] for corroborating the proposed HCRF model performance. All the datasets are explained below.

#### 4.1.1. Weizmann Action Dataset

This dataset consisted of 10 actions such as bending, running, walking, skipping, place jumping, side movement, jumping forward, two hand waving, and one hand waving that were performed by total 9 subjects. This dataset comprised of 90 video clips with average of 15 frames per clip where the frame size is 144 × 180.

#### 4.1.2. KTH Action Dataset

KTH dataset employed for activity recognition comprised of 25 subjects who performed 6 activities like running, walking, boxing, jogging, handclapping, and hand waving in four distinctive scenarios. Using a static camera, in the homogenous background, a total of 2391 sequences were taken with a frame size of 160 × 120.

#### 4.1.3. UCF Sports Dataset

In this dataset, there were 182 videos which were evaluated by *n*-fold cross-validation rule. This dataset has been taken from different sports activities in broadcast television channels. Some of the videos had high intraclass similarities. This dataset was also collected using a static camera. This dataset covers 9 activities like running, diving, lifting, golf swinging, skating, kicking, walking, horseback riding, and baseball swinging. Each frame has a size of 720 × 480.

#### 4.1.4. IXMAS Action Dataset

IXMAS (INRIA Xmas motion acquisition sequences) dataset comprised of 13 activity classes which were performed by 11 actors, each 3 times. Every actor opted a free orientation as well as position. The dataset has provided annotated silhouettes for each person. For our experiments, we have selected only 8 action classes like walk, cross arms, punch, turn around, sit down, wave, get up, and kick. IXMAS dataset is a multiview dataset for a view-invariant human activity recognition where each frame has a size of 390 × 291. This dataset has a major occlusion and that may cause misclassification; therefore, we utilized global histogram equalization [61] in order to resolve the occlusion issue.

### 4.2. Setup

For a comprehensive validation, we carried out the following set of experiments executed using Matlab.

- The first experiment was conducted on each dataset separately in order to show the performance of the proposed model. In this experiment, we employed 10-fold cross-validation rule, which means that data from 9 subjects were utilized for training data, while the data from one subject was picked as a testing data. The procedure was reiterated for 10 times provided each subject data is utilized for both training and testing.
- The second experiment was conducted in the absence of the proposed recognition model on all the four datasets that will show the importance of the developed model. For this purpose, we used the existing eminent classifiers like SVM, ANN, HMM, and existing HCRF [30] as a recognition model rather than utilizing the proposed HCRF model.
- The third experiment was conducted to show the performance of the proposed approach against the state-of-the-art methods.
- In the last experiment, the computational complexity of the proposed HCRF model was compared with forward/backward algorithms.

## 5. Results and Discussion

### 5.1. First Experiment

As described before, this experiment validates the performance of the proposed recognition model on an individual dataset. The overall results are shown in Tables 1 (using Weizmann dataset), 2 (using KTH dataset), 3 (using UCF sports dataset), and 4 (using IXMAS), respectively.

As observed from Tables 1 [Table tab2] [Table tab3]–4, the proposed recognition model constantly obtained higher recognition rates on individual dataset. This result shows the robustness of the proposed model which means that the model not only showed better performance on one dataset but also showed better performance across multiple spontaneous datasets.

### 5.2. Second Experiment

As described before, the second experiment was conducted in the absence of the proposed recognition model, to show the importance of the proposed model using all the four datasets. For this purpose, we used the existing eminent classifiers like SVM, ANN, HMM, and existing HCRF [30] as a recognition model rather than utilizing the proposed HCRF model.

Tables 5 [Table tab6] [Table tab7]–8 show that when the proposed HCRF model was substituted with ANN, SVM, HMM, and existing HCRF [30], the system failed to accomplish higher recognition rates. The better performance of the proposed HCRF model is visualized in Tables 1 [Table tab2] [Table tab3]–4, which show that the proposed HCRF model effectively fix the drawbacks of HMM and existing HCRF that has been extensively utilized for sequential HAR.

### 5.3. Third Experiment

In this experiment, a comparative analysis was made between the state-of-the-art methods and the proposed model. All of these approaches were implemented by the instructions provided in their particular articles. A 10-fold cross-validation rule was employed on each dataset as explained in Section 4. The average classification results of the existing methods along with the proposed method across different datasets are summarized in Table 9.

It is obvious from Table 9 that the proposed method showed a significant performance against the existing state-of-the-art methods. Therefore, the proposed method accurately and robustly recognizes the human activities using different video data.

### 5.4. Fourth Experiment

In this experiment, we have presented the computational complexity that is also one of the contributions in this paper. The implementations of the previous HCRF are available in literature, which calculate the gradients by reiterating the forward and backward techniques, while the proposed HCRF model executes them once only and cashes the outcomes for the later use. From (21) and (22), it is clear that the forward or backward technique has a complexity of *O*(*TQ* ^2^ *M*), where *T* represents the input sequence length, *Q* represents the number of states, and *M* indicates the number of mixtures. The proposed HCRF model, however, requires a full complexity of *O*(*TM*) to calculate gradients as can be seen from (22)–(29).

**Figure 3:**
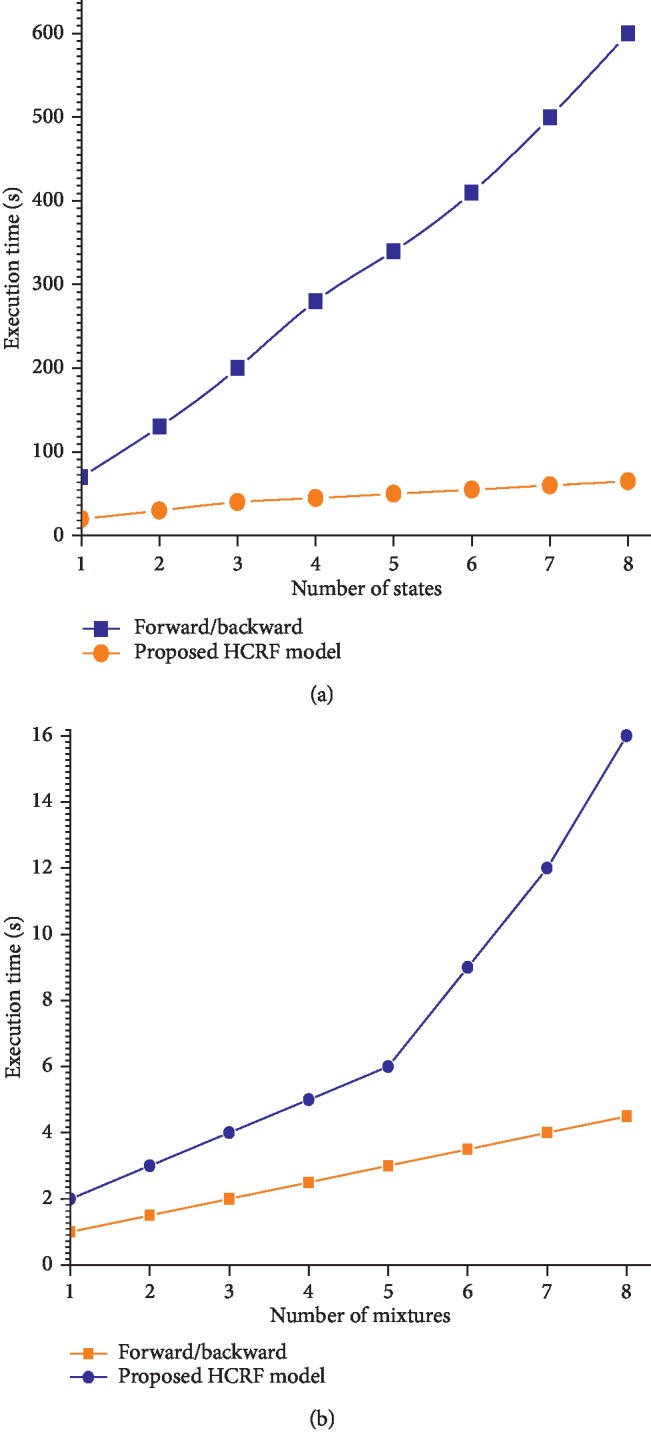
An illustration of gradient computational time (equation (30)) of the previous forward and backward algorithms and the proposed HCRF model. (a) *Q*=1 − 5, *M*=5, *T*=90 and (b), *Q*=5, *M*=1 − 5, *T*=90.

Figure 3 shows a comparison of the execution time when the gradients are computed by the forward (or backward) algorithm and by our proposed method. The computational time is calculated by running Matlab R2013a with the specification of Intel® Pentium® Core™ i7-6700 (3.4 GHz) with a RAM capacity of 16 GB.

## 6. Conclusion

In healthcare and telemedicine, the human activity recognition (HAR) can be best explained by helping physically disable persons' scenario. A paralyzed patient with half of the body critically attacked by paralysis is completely unable to perform their daily exercises. The doctors recommend specific activities to get better improvement in their health. So, for this purpose, the doctors need a human activity recognition (HAR) system through which they can monitor the patients' daily routines (activities) on a regular basis.

The accuracy of most of the HAR systems depends upon the recognition modules. For feature extraction and selection modules, we used some of the existing well-known methods, while for the recognition module, we proposed the usage of HCRF model which is capable of approximating a complex distribution using a mixture of Gaussian density functions. The proposed model was assessed against four publicly available standard action datasets. From our experiments, it is obvious that the proposed full-covariance Gaussian density function showed a significant improvement in accuracy than the existing state-of-the-art methods. Furthermore, we also proved that such improvement is significant from statistical point of view by showing value ≤0.2 of the comparison. Similarly, the complexity analysis points out that the proposed computational method strongly decreases the execution time for the hidden conditional random field model.

The ultimate goal of this study is to deploy the proposed model on smartphones. Currently, the proposed model is using full-covariance matrix; however, this might be time consuming, especially when using on smartphones. Using a lightweight classifier such as K-nearest neighbor (K-NN) could be one possible solution. But K-NN is very much sensitive to environmental factor (like noise). Therefore, in future, we will try to investigate further research to reduce the time and sustain the same recognition rate when employing on smartphones in real environment.

## Figures and Tables

**Table 1:** Confusion matrix of the proposed recognition model using Weizmann action dataset (unit: %).

Activities	Bend	Jack	Pjump	Run	Side	Skip	Walk	Wave 1	Wave 2
Bend	**98**	0	1	0	0	1	0	0	0
Jack	1	**96**	0	0	2	0	1	0	0
Pjump	0	1	**97**	0	1	0	0	1	0
Run	0	0	0	**99**	0	0	0	1	0
Side	1	2	0	0	**95**	0	0	2	0
Skip	0	0	0	0	0	**100**	0	0	0
Walk	1	0	0	1	0	1	**96**	0	1
Wave 1	0	1	1	0	0	0	0	**98**	0
Wave 2	0	1	0	0	1	1	1	1	**95**
Average	**97.11**								

**Table 2 tab2:** Confusion matrix of the proposed recognition model using KTH action dataset (unit: %).

Activities	Walking	Jogging	Running	Boxing	Hand-wave	Handclap
Walking	**100**	0	0	0	0	0
Jogging	0	**98**	1	1	0	0
Running	2	1	**95**	1	1	0
Boxing	0	2	1	**97**	0	0
Hand-wave	1	0	1	0	**98**	0
Handclap	0	0	1	0	0	**99**
Average	**97.83**					

**Table 3 tab3:** Confusion matrix of the proposed recognition model using UCF sports dataset (unit: %).

Activities	Diving	GS	Kicking	Lifting	HBR	Run	Skating	BS	Walk
Diving	**95**	1	2	1	0	1	0	0	0
GS	1	**94**	0	0	2	1	1	1	0
Kicking	0	2	**98**	0	0	0	0	0	0
Lifting	1	1	1	**94**	1	0	0	1	1
HBR	0	0	2	0	**96**	1	0	1	0
Running	0	3	0	0	0	**97**	0	0	0
Skating	1	0	1	1	0	1	**95**	0	1
BS	0	0	0	1	0	1	0	**97**	1
Walking	0	0	0	0	0	0	0	0	**100**
Average	**96.22**								

GS: golf swinging, HBR: horseback riding, and BS: baseball swinging.

**Table 4:** Confusion matrix of the proposed recognition model using IXMAS action dataset (unit: %).

Activities	CA	SD	GU	TA	Walk	Wave	Punch	Kick
CA	**97**	0	0	1	2	0	0	0
SD	0	**99**	1	0	0	0	0	0
GU	1	2	**94**	3	0	0	0	0
TA	0	0	1	**95**	2	1	1	0
Walk	0	1	1	0	**98**	0	0	0
Wave	0	0	2	0	1	**97**	0	0
Punch	0	1	0	1	0	2	**96**	0
Kick	0	0	0	0	1	0	0	**99**
Average	**96.88**							

CA: cross arm, SD: sit down, GU: get up, and TA: turn around.

**Table 5:** Classification results of the proposed system on Weizmann action dataset (A) using ANN, (B) using SVM, (C) using HMM, and (D) using existing HCRF [30], while removing the proposed HCRF model (unit: %).

Activities	Bend	Jack	Pjump	Run	Side	Skip	Walk	Wave 1	Wave 2
*(A)*									
Bend	**70**	4	5	3	3	2	5	5	3
Jack	4	**68**	3	6	7	2	3	3	4
Pjump	2	4	**75**	6	3	2	2	2	4
Run	4	2	3	**72**	6	2	5	3	3
Side	5	3	5	4	**65**	6	4	6	2
Skip	4	6	4	3	5	**67**	3	2	6
Walk	4	2	4	7	3	4	**70**	3	3
Wave 1	2	1	3	3	4	5	7	**71**	4
Wave 2	2	5	3	6	4	4	3	5	**68**
Average	**69.55**								
*(B)*									
Bend	**69**	3	4	4	6	4	2	3	5
Jack	2	**72**	2	3	4	3	5	4	5
Pjump	1	4	**75**	2	4	5	4	2	3
Run	2	4	3	**78**	2	2	4	2	3
Side	2	4	5	3	**70**	4	3	5	4
Skip	2	1	3	2	4	**80**	3	3	2
Walk	2	0	3	4	3	2	**82**	1	3
Wave 1	2	2	3	4	3	2	3	**77**	4
Wave 2	1	2	1	2	3	1	3	4	**83**
Average	**76.22**								
*(C)*									
Bend	**82**	3	0	2	2	3	1	5	2
Jack	3	**80**	1	2	3	2	3	4	2
Pjump	3	4	**85**	3	0	0	1	2	2
Run	5	4	2	**79**	0	2	1	3	4
Side	0	1	5	4	**81**	3	1	2	3
Skip	3	1	2	2	3	**88**	0	0	1
Walk	0	2	3	2	1	2	**83**	3	4
Wave 1	1	3	2	2	4	2	3	**78**	5
Wave 2	1	2	2	2	2	3	1	0	**87**
Average	**82.56**								
*(D)*									
Bend	**80**	2	3	1	4	0	5	2	3
Jack	1	**88**	0	2	0	3	2	3	1
Pjump	0	2	**90**	1	0	3	0	2	2
Run	2	1	2	**85**	2	3	0	0	5
Side	4	1	2	3	**80**	4	1	2	3
Skip	1	4	0	5	1	**84**	0	3	2
Walk	2	1	0	0	1	2	**89**	2	3
Wave 1	3	0	1	2	0	2	0	**91**	1
Wave 2	4	1	3	0	2	3	0	2	**85**
Average	**85.78**								

**Table 6 tab6:** Classification results of the proposed system on KTH action dataset (A) using ANN, (B) using SVM, (C) using HMM, and (D) using existing HCRF [30], while removing the proposed HCRF model (unit: %).

Activities	Walking	Jogging	Running	Boxing	Hand-wave	Handclap
*(A)*						
Walking	**79**	5	6	4	3	3
Jogging	3	**81**	5	3	4	4
Running	6	4	**77**	5	5	3
Boxing	6	7	6	**69**	5	7
Hand-wave	4	7	5	5	**73**	6
Handclap	4	6	5	4	6	**75**
Average	**75.66**					
*(B)*						
Walking	**82**	2	3	5	4	4
Jogging	3	**86**	2	3	2	4
Running	5	3	**80**	4	5	3
Boxing	5	3	3	**79**	4	6
Hand-wave	1	4	3	3	**89**	0
Handclap	3	5	2	4	3	**83**
Average	**83.17**					
*(C)*						
Walking	**86**	3	2	4	2	3
Jogging	0	**88**	3	2	4	3
Running	0	3	**90**	0	4	3
Boxing	3	0	4	**92**	1	0
Hand-wave	1	3	2	2	**91**	1
Handclap	1	3	4	1	2	**89**
Average	**89.33**					
*(D)*						
Walking	**90**	3	0	3	4	0
Jogging	2	**88**	2	3	3	2
Running	4	2	**92**	0	0	2
Boxing	1	3	2	**91**	3	0
Hand-wave	0	1	3	2	**93**	1
Handclap	1	3	2	4	3	**87**
Average	**90.17**					

**Table 7 tab7:** Classification results of the proposed system on UCF sports dataset (A) using ANN, (B) using SVM, (C) using HMM, and (D) using existing HCRF [30], while removing the proposed HCRF model (unit: %).

Activities	Diving	GS	Kicking	Lifting	HBR	Run	Skating	BS	Walk
*(A)*									
Diving	**68**	4	2	5	6	6	4	3	2
GS	2	**71**	2	4	5	4	6	3	3
Kicking	3	4	**70**	3	5	4	2	3	6
Lifting	5	4	3	**65**	5	6	4	6	2
HBR	3	4	6	3	**66**	4	5	5	4
Running	3	3	5	4	6	**64**	6	4	5
Skating	2	5	4	5	3	4	**69**	3	5
BS	4	2	5	3	4	6	5	**67**	4
Walking	5	4	2	3	4	3	6	3	**70**
Average	**67.78**								
*(B)*									
Diving	**71**	4	2	3	5	6	3	2	4
GS	3	**77**	2	4	3	2	5	2	2
Kicking	4	2	**74**	4	5	3	2	3	3
Lifting	5	6	3	**69**	4	3	5	3	2
HBR	2	3	3	2	**80**	2	4	2	2
Running	2	3	2	2	5	**75**	6	2	3
Skating	2	1	2	3	4	4	**78**	2	4
BS	3	4	6	3	4	2	3	**70**	5
Walking	4	1	2	4	2	3	0	3	**81**
Average	**75.00**								
*(C)*									
Diving	**79**	3	2	2	3	4	3	2	2
GS	0	**83**	2	4	3	2	1	3	2
Kicking	1	2	**85**	1	3	3	2	3	0
Lifting	3	0	2	**82**	3	2	4	2	2
HBR	0	2	2	4	**80**	0	5	3	4
Running	1	2	1	3	4	**84**	2	1	2
Skating	2	0	3	4	0	1	**86**	3	1
BS	1	1	1	2	0	3	0	**88**	4
Walking	1	2	4	2	5	2	4	3	**77**
Average	**82.67**								
*(D)*									
Diving	**90**	3	0	1	0	2	2	1	1
GS	3	**84**	2	1	3	1	3	2	1
Kicking	3	4	**85**	0	0	2	3	1	2
Lifting	1	2	1	**89**	1	1	1	2	2
HBR	0	2	1	0	**91**	2	3	0	1
Running	2	3	1	2	3	**80**	4	2	3
Skating	2	4	1	2	3	0	**84**	4	0
BS	2	1	1	2	1	0	3	**88**	2
Walking	0	2	1	1	0	1	4	0	**91**
Average	**86.89**								

GS: golf swinging, HBR: horseback riding, BS: baseball swinging.

**Table 8:** Classification results of the proposed system on IXMAS action dataset (A) using ANN, (B) using SVM, (C) using HMM, and (D) using existing HCRF [30], while removing the proposed HCRF model (unit: %).

Activities	CA	SD	GU	TA	Walk	Wave	Punch	Kick
*(A)*								
CA	**65**	5	7	6	5	4	3	5
SD	5	**72**	4	3	5	4	3	4
GU	4	3	**75**	5	3	2	4	4
TA	6	7	4	**68**	3	5	4	3
Walk	3	4	5	4	**70**	6	4	4
Wave	4	6	5	3	4	**71**	3	4
Punch	3	5	5	6	7	3	**67**	4
Kick	4	5	4	6	5	4	3	**69**
Average	**69.62**							
*(B)*								
CA	**77**	3	4	2	2	3	5	4
SD	3	**79**	3	2	4	5	2	2
GU	5	6	**69**	3	4	5	4	4
TA	2	3	2	**80**	4	4	3	2
Walk	3	5	4	2	**71**	5	6	4
Wave	2	6	3	5	4	**73**	4	3
Punch	1	5	3	4	1	3	**81**	2
Kick	3	6	7	4	3	5	4	**68**
Average	**74.75**							
*(C)*								
CA	**79**	3	4	1	2	4	3	4
SD	1	**84**	3	2	3	1	4	2
GU	0	1	**88**	1	2	3	2	3
TA	5	2	3	**79**	2	3	2	4
Walk	1	0	3	1	**90**	2	3	0
Wave	2	3	1	0	3	**86**	2	3
Punch	1	0	2	3	0	4	**89**	1
Kick	3	2	4	1	0	2	4	**84**
Average	**84.77**							
*(D)*								
CA	**90**	1	2	0	3	4	0	0
SD	3	**85**	2	1	3	3	2	1
GU	0	1	**91**	1	0	2	3	2
TA	1	3	2	**87**	1	2	2	2
Walk	1	0	3	1	**89**	2	1	3
Wave	0	2	1	0	2	**90**	3	1
Punch	1	4	2	1	2	3	**84**	3
Kick	1	2	4	1	3	2	4	**83**
Average	**87.38**							

CA: cross arm, SD: sit down, GU: get up, TA: turn around.

**Table 9:** Weighted average recognition rates of the proposed method with the existing state-of-the-art methods (unit: %).

State-of-the-art works	Average classification rates	Standard deviation
GMM	63.3	±2.7
SVM	67.5	±4.4
HMM	82.8	±3.8
Embedded HMM	85.9	±1.9
[62]	92.1	±3.2
[63]	84.3	±4.9
[18]	93.6	±2.7
[19]	93.0	±1.6
[22]	92.7	±2.5
[64]	80.1	±3.2
Proposed method	**97.2**	±2.8

## Data Availability

The data used to support the findings of this study are available from the corresponding author upon request.
